# *Nodularia spumigena* Peptides—Accumulation and Effect on Aquatic Invertebrates

**DOI:** 10.3390/toxins7114404

**Published:** 2015-10-30

**Authors:** Hanna Mazur-Marzec, Katarzyna Sutryk, Agnieszka Hebel, Natalia Hohlfeld, Anna Pietrasik, Agata Błaszczyk

**Affiliations:** Department of Marine Biotechnology, Institute of Oceanography, University of Gdańsk, Al. Marszałka Piłsudskiego 46, 81-378 Gdynia, Poland; E-Mails: biohm@ug.edu.pl (H.M.-M.); katarzyna.sutryk@gmail.com (K.S.); agnieszkam.hebel@gmail.com (A.H.); hohlfeld@poczta.fm (N.H.); anna.pietrasik@ug.edu.pl (A.P); agata.blaszczyk@ug.edu.pl (A.B.)

**Keywords:** bioaccumulation, blue mussels, Baltic cyanobacteria, cyanopeptides, crustaceans, *Nodularia spumigena*

## Abstract

Thus far, the negative effects of *Nodularia spumigena* blooms on aquatic organisms have been mainly attributed to the production of the hepatotoxic nodularin (NOD). In the current work, the accumulation of other *N. spumigena* metabolites in blue mussels and crustaceans, and their effect on *Thamnocephalus platyurus* and *Artemia franciscana,* were examined. The liquid chromatography-tandem mass spectrometry (LC-MS/MS) analyses provided evidence that both blue mussels collected after a cyanobacterial bloom in the Baltic Sea and the crustaceans exposed under laboratory conditions to *N. spumigena* extract accumulated the cyclic anabaenopeptins (APs). In the crustaceans, the linear peptides, spumigins (SPUs) and aeruginosins (AERs), were additionally detected. Exposure of *T. platyurus* and *A. franciscana* to *N. spumigena* extract confirmed the negative effect of nodularin on the organisms. However, high numbers of dead crustaceans were also recorded in the nodularin-free fraction, which contained protease inhibitors classified to spumigins and aeruginosins. These findings indicate that cyanobacterial toxicity to aquatic organisms is a complex phenomenon and the induced effects can be attributed to diverse metabolites, not only to the known hepatotoxins.

## 1. Introduction

In fresh, marine, and brackish waters throughout the world, anthropogenic eutrophication, together with favorable weather conditions, have a stimulating effect on cyanobacterial growth and bloom formation [1,2]. The ecological consequences of the blooms include changes in the physical and chemical conditions of aquatic ecosystems and the direct and indirect impact of cyanobacteria, especially toxic species, on other aquatic organisms. Generally, invertebrates were shown to be less affected by cyanobacteria than fish [3,4]. Within this group of organisms, however, intraspecies differences in the reactions were observed [5,6].

In the Baltic Sea, the cyanobacterial community is composed of the filamentous and diazotrophic representatives of the Nostocales order, *Nodularia spumigena*, *Aphanizomenon flos-aquae* and *Dolichospermum* spp., and the single-celled picocyanobacteria, mainly from the genus *Synechococcus* (Chroococcales order) [7,8]. Although the filamentous species were thought to be difficult to assimilate by aquatic herbivores [9], recent studies documented their significance as a supplementary food for Baltic copepods and mysids. Some authors showed positive effects of cyanobacteria on zooplankton reproduction [10,11,12], while contradictory results were published by other researchers [13,14,15,16]. According to Engström-Öst *et al.* [14], the content of the toxic *N. spumigena* in the guts of *Acartia* was negatively related to egg production and the condition of the female copepod.

The interaction between cyanobacteria and their potential grazers is presumed to be, at least partially, mediated by bioactive cyanobacterial metabolites, including toxins. The compounds might play the role of signaling molecules or defense agents against grazers [17,18,19,20,21,22,23]. The hepatotoxic cyclic peptides, nodularins and microcystins, belong to the most frequently occurring and most widely studied cyanobacterial metabolites. Their effect and accumulation in aquatic animals from different trophic levels were documented, both in field studies and in laboratory experiments [3,4,24,25,26,27,28].

There is accumulating evidence that negative consequences of toxic cyanobacteria on aquatic invertebrates, if observed, can also be attributed to cyanobacterial metabolites other than the cyclic hepatotoxic peptides, nodularin or microcystins. Some of the peptides inhibit the activity of important metabolic enzymes, including proteases and protein phosphatases [29,30,31,32,33,34]. Inhibitors of these enzymes are also produced by the Baltic cyanobacteria, including *N. spumigena* [35].

To gain a better understanding about the impact of cyanobacteria and their metabolites on other organisms, different toxicological tests have been performed. In preliminary assessments of cyanobacterial toxicity, the commercially available biotests on commonly occurring zooplankton crustaceans have been frequently used [5,36]. The tests constitute an attractive alternative, free from ethical constrains, for bioassays performed on vertebrates [37,38].

The aim of the present work was to determine which cyanobacteria metabolites produced by the Baltic filamentous cyanobacterium *N. spumigena* accumulate in blue mussels and crustaceans. For the purpose of the study, mussels collected in the area of cyanobacterial blooms in the Gulf of Gdańsk, southern Baltic Sea, were analyzed. The accumulation of *N. spumigena* peptides in two anostracan crustaceans, *Thamnocephalus platyurus* and *Artemia franciscana,* was additionally tested under laboratory conditions. Finally, to assess the effect of the accumulated metabolites on the invertebrates, microbiotests with the two crustaceans exposed to a crude extract, fractions, and spent medium from *N. spumigena* culture were conducted.

## 2. Results

### 2.1. Accumulation of Cyanopeptides in Crustaceans (Laboratory Experiment) and in Blue Mussels (Environmental Samples)

In the Gulf of Gdańsk, the highest biomass of filamentous cyanobacteria was recorded in July. With the exception of four cyclic hexapeptides classified to anabaenopeptins (APs) with mass to charge ratio, *m/z*, 918, 916, 837 and 824 and one spumigin (SPU) with *m/z* 627, the compounds included in Table 1 were detected in all phytoplankton samples. In August, the concentrations of the peptides were below the detection limit of liquid chromatography-tandem mass spectrometry (LC-MS/MS, 0.5–1.5 sng mL^−1^).

The LC-MS/MS analysis of mussels collected from the Gulf of Gdańsk revealed the presence of nodularin (NOD) and nine anabaenopeptins (Table 1). The compounds were detected using MRM mode with transitions specific for individual peptides. Subsequently, the structures of the peptides were confirmed based on the fragmentation spectra of their pseudomolecular ions [M+H]^+^ (Figure S1–S4). Similar to the bloom sample, for both classes of mussels, the area of the NOD ion peak was the largest. The signals produced by AP variants with [M+H]^+^ ions at *m/z* 868, 851, 824, and 808 were also strong. In the case of bigger mussels (*L* > 3), the area of APs peaks in MRM chromatograms were within the range 0.5 × 10^7^–1.7×10^7^ and were always slightly smaller than the areas of the same AP peaks detected in smaller mussels L < 2 cm (0.7×10^7^–1.9×10^7^). Two anabaenopeptins, with *m/z* 902 (0.3 × 10^7^) and *m/z* 828 (0.9×10^7^), were present only in mussels with shell lengths below 2 cm. Neither of the linear tetrapeptides, spumigins, or aeruginosins (AERs), were detected in soft tissue of the mussels.

**Figure 1:**
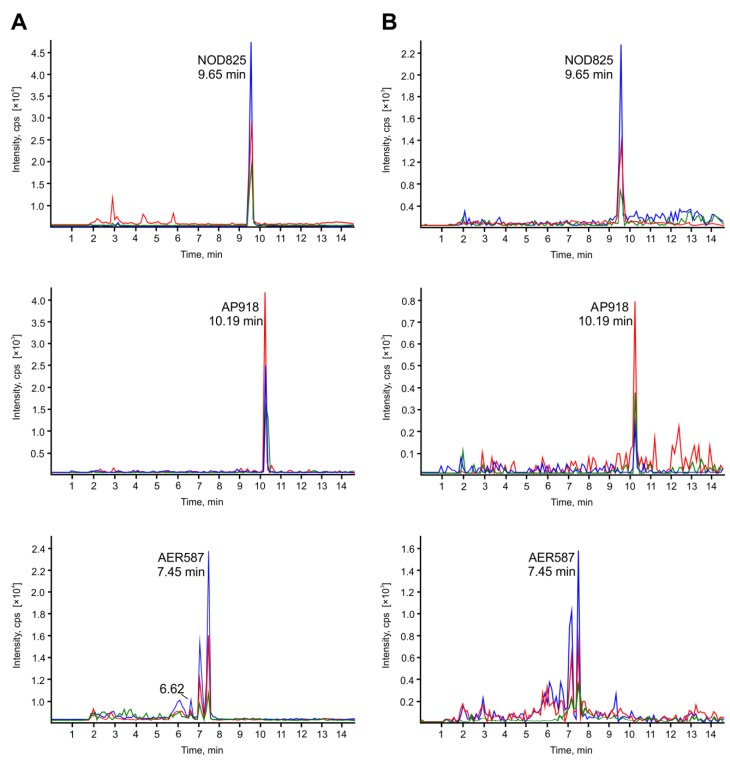
MRM chromatograms for nodularin NOD825, anabaenopeptin AP918 and aeruginosin AER587 in *Artemia franciscana* (**A**) and *Thamnocephalus platyurus* (**B**) exposed to *Nodularia spumigena* extracts.

In *A. franciscana* and *T. platyurus* exposed for 24 h to cyanobacterial extract, representatives of four classes of peptides were found (Table 1). In the mass spectra, the area of the NOD peak was the largest. The crustaceans also accumulated 6–8 variants of anabaenopeptins, one aeruginosin (*m/z* 587), and one spumigin (*m/z* 611) (Figure S5). The peak areas in the MRM chromatograms of peptides from *A. franciscana* extract were always larger than for corresponding peaks in the chromatograms from *T. platyurus* extract (Table 1, Figure 1).

**Table 1:** Cyanobacterial peptides identified in *T. platyurus*, *A. franciscana* (laboratory experiment), blue mussels, and bloom material (environmental samples from the Baltic Sea) and peak areas of their selected transitions in MRM chromatogram recalculated per 100 mg of dry biomass.

*m/z* [M+H]^+^	Peptide structure	Peak area (counts)				
		Laboratory experiment		Environmental samples		
		*A. franciscana* 100 mg d.w.	*T. platyurus* 100 mg d.w.	Blue mussels 100 mg d.w. 20 August 2012		Cyanobacteria 100 mg d.w. 27 July 2012
				*L* > 3 cm	*L* < 2 cm	
Cyclic peptides: anabaenopeptins, nodularin						
AP 918	Phe-CO-[Lys-Val-Hph-MeHty-MetO]	1.1 × 10^5^	0.6 × 10^5^	0.5 × 10^7^	0.7 × 10^7^	0.9 × 10^7^
AP 916	Phe-CO-[Lys-Val-Hty-MeHty-AcSer]	-	Traces	-	-	0.3 × 10^7^
AP 902	Phe-CO-[Lys-Val-Hph-MeHty-Met]	0.9 × 10^5^	0.2 × 10^5^	-	0.3 × 10^7^	0.4 × 10^7^
AP 900	Phe-CO-[Lys-Val-Hph-MeHty-AcSer]	1.3 × 10^5^	0.26 × 10^5^	-	-	0.5 × 10^7^
AP 884	Ile-CO-[Lys-Val-Hph-MeHty-MetO]	3.2 × 10^5^	1.8 × 10^5^	0.8 × 10^7^	0.9 × 10^7^	1.2 × 10^7^
AP 868	Ile-CO-[Lys-MetO-Hty-MeHty-Met]	-	Traces	1.7 × 10^7^	1.9 × 10^7^	1.5 × 10^7^
AP 851 (E)	Arg-CO-[Lys-Val-Hty-MeAla-Phe]	13.5 × 10^5^	3.8 × 10^5^	1.4 × 10^7^	1.5 × 10^7^	2.1 × 10^7^
AP 837 (B)	Arg-CO-[Lys-Val-Hty-MeAla-Phe]	-	-	0.9 × 10^7^	1.0 × 10^7^	0.5 × 10^7^
AP 828	Phe-CO-[Lys-Val-Hty-MeAla-Phe]	22.9 × 10^5^	5.9 × 10^5^	-	0.9 × 10^7^	1.7 × 10^7^
AP 824	Ile-CO-[Lys-Val-Hph-MeHty-Ser]	-	-	1.2 × 10^7^	1.4 × 10^7^	5.6 × 10^7^
AP 808	Ile-CO-[Lys-Val-Hty-MeAla-Hph ]	-	-	1.3 × 10^7^	1.5 × 10^7^	1.5 × 10^7^
NOD 825	Cyclo[MeAsp-Arg-Adda-Glu-Mdhb]	16.0 × 10^6^	1.1 × 10^6^	2.6 × 10^7^	2.8 × 10^7^	5.7 × 10^7^
[Asp]^3^NOD811	Cyclo[Asp-Arg-Adda-Glu-Mdhb]	-	-	1.3 × 10^7^	1.5 × 10^7^	3.2 × 10^7^
Linear peptides: spumigins, aeruginosin						
SPU 627	Hpla-Hty-MePro-Arg	-	-	-	-	0.2 × 10^7^
SPU 613	Hpla-Hty-MePro-Argol	-	-	-	-	0.3 × 10^4^
SPU 611	Hpla-Hty-MePro-Argal	7.1 × 10^6^	0.6 × 10^5^	-	-	2.5 × 10^7^
SPU 597	Hpla-Hty-Pro-Argal	-	-	-	-	0.2 × 10^4^
AER 587	HA-Tyr-Choi-Argal	0.2 × 10^6^	0.1 × 10^6^	-	-	1.1 × 10^7^

AER—aeruginosin, AP—anabaenopeptin, NOD—nodularin, SPU—spumigin, HA—hexanoic acid, Hpla—(4-hydroxy-phenyl) lactic acid, Hph—homophenylalanine, Hty—homotyrosine).

### 2.2. Effect of N. spumigena Spent Medium and Cell Extracts on Crustaceans

**Figure 2:**
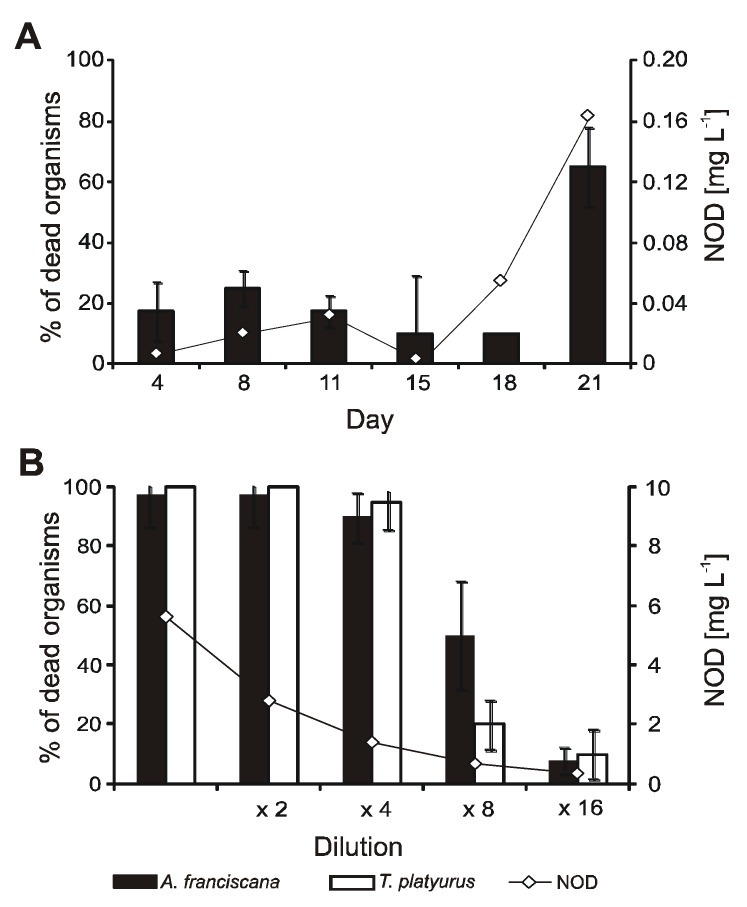
Mortality of *A. franciscana* and *T. platyurus,* exposed to *N. spumigena* spent media collected during growth of the culture (**A**) and to *N. spumigena* cell extract collected on 21 day of the culture (**B**). Nodularin (NOD) concentrations in the samples are marked with a diamond.

The media collected on days 4, 8, 11, 15, 18, and 21 of *N. spumigena* culture had no significant effect on the crustaceans. The increased mean mortality of *A. franciscana* was evoked by the sample from the late exponential stage of *N. spumigena* growth (Figure 2A). In the medium collected on day 21, 57% of organisms died. During 24-hour exposure, *T. platyurus* did not react to the spent medium from *N. spumigena* culture. The concentration of NOD in the spent medium increased from 0.007 mg∙L^−1^ on the first day of culture to 0.16 mg∙L^−1^ on day 21. In the medium collected in the late exponential phase of growth, two anabaenopeptins with *m/z* 842 (peak area 2.5 × 10^6^) and *m/z* 828 (peak area 1.3 × 10^6^), one spumigin with *m/z* 597 (peak area 1.1 × 10^6^), and one aeruginosin with *m/z* 587 (peak area 1.5 × 10^5^) were also detected.

The effect of *N. spumigena* cell extract on both crustacean species was similar and depended on the concentration of the sample (Figure 2B). The highest concentration of NOD in the tested extract was 5.6 mg∙L^−1^. The three most concentrated samples caused death of over 90% of the organisms. In the eight times diluted cyanobacterial extract, *ca.* 50% mortality of *A. franciscana*, and 25% mortality of *T. platyurus* was recorded (Figure 2B). In the most diluted extracts, only 8% of *A. franciscana* and 10% of *T. platyurus* died.

### 2.3. Effect of Individual Fractions Obtained from N. spumigena Cell Extracts on A. franciscana

The biological activity of the four fractions obtained from *N. spumigena* cell extracts was tested using *A. franciscana*. These four fractions were also analyzed by LC-MS/MS. Structure elucidation of the peptides present in the fractions was based on the *m/z* (mass to charge) values and the fragmentation spectra of their pseudomolecular ions [M+H]^+^. The composition of the four fractions is shown in Table 2.

**Figure 3:**
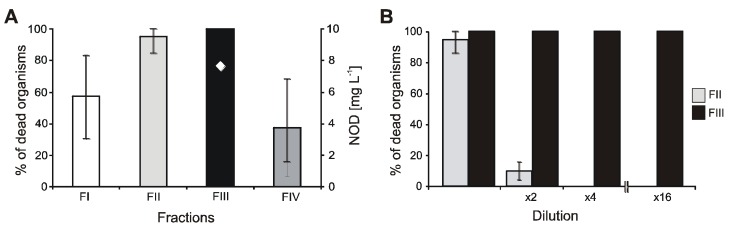
Mortality percentage of *A. franciscana* exposed to cell extract from *N. spumigena* CCNP1401 (**A**) and to diluted fractions II and III (**B**).

In undiluted fraction III, which contained NOD (7.6 mg∙L^−1^), a demethylated form of the toxin, and small amounts of two APs (Table 2), all test organisms died (Figure 3A). The mortality of crustaceans did not diminish even in the 16-times diluted solution (Figure 3B). In fraction II, six spumigins and three aeruginosins were detected. The mortality of the crustaceans incubated in the presence of these compounds was also high (90%), however, it dropped to 10% (no toxic effects) in the 2-times diluted sample (Figure 3B). Fraction I, which contained four spumigins, was less toxic (57.5% mortality). The lowest mean mortality of *A. franciscana* (37.5%) was in fraction IV in which trace amounts of NOD were found and two anabaenopeptins were detected as the main components (Table 2).

**Table 2:** Composition of fractions obtained from *N. spumigena* CCNP1401 cell extract.

HPLC fraction	I		II		III		IV	
Retention time [min]	0.0–3.0		3.1–7.7		7.8–10.0		10.1–15.0	
Peptides identified in fractions	*m/z* [M+H]^+^	Ion peak area	*m/z* [M+H]^+^	Ion peak area	*m/z* [M+H]^+^	Ion peak area	*m/z* [M+H]^+^	Ion peak area
	SPU 641	2.0 × 10^7^	SPU 653	6.0 × 10^6^	AP 932	5.0 × 10^6^	AP 842	3.0 × 10^7^
	SPU 639	2.0 × 10^7^	SPU 639	3.0 × 10^7^	AP 872	6.0 × 10^6^	AP 828	3.0 × 10^7^
	SPU 597	3.5 × 10^7^	SPU 627	8.0 × 10^6^	NOD 825	3.2 × 10^7^	NOD 825	1.5 × 10^6^
	SPU 599	3.3 × 10^7^	SPU 613	1.1 × 10^7^	[Asp^3^]NOD 811	2.8 × 10^7^		
			SPU 611	1.5 × 10^7^				
			SPU 597	3.4 × 10^7^				
			AER 595	1.2 × 10^7^				
			AER 589	4.0 × 10^6^				
			AER 587	1.4 × 10^7^				

AER: aeruginosin; AP: anabaenopeptin; NOD: nodularin; SPU: spumigin.

## 3. Discussion

### 3.1. Accumulation of Cyanobacteria Metabolites in Aquatic Invertebrates

Aquatic invertebrates are mainly exposed to cyanobacterial toxins through the consumption of contaminated food, but direct uptake of the compounds from water was also documented [39]. In water, the concentrations of microcystins, nodularins, and probably many other metabolites, increase during the later stages of cyanobacterial growth and reach their maxima after cell lyses and death [40]. Also in our work, the increase in NOD concentration in the medium during the three-week *N. spumigena* culture was observed. This change in toxin concentration corresponded to a higher number of dead *A. franciscana* organisms in spent medium collected at the end of the culture growth. As in the medium from the culture in its late exponential phase of growth, two anabaenopeptins (*m/z* 842, 828), one spumigin (*m/z* 597) and one aeruginosin (587) were also detected; the additive effect of these peptides on tested organisms should be considered. Accumulation of nodularin in aquatic biota has been studied by many research groups. In the Baltic, during a bloom event, NOD concentrations in zooplankton and blue mussels reached 0.62 μg∙g^−1^ d.w. and 2.15 μg∙g^−1^ d.w., respectively [3,8,41,42]. In blue mussels from the Gulf of Gdańsk, high concentrations of the toxin were measured even in late October, over three months after the *N. spumigena* peak [8]. Under laboratory exposure, invertebrates accumulated 4.94 μg NOD per g d.w. and 80.4 μg NOD per g d.w., at the maximum [39,43].

Apart from nodularin and microcystins, cyanobacteria produce many other bioactive peptides [32,34]; however, the knowledge about their accumulation is limited to one report. In fauna from Greek freshwaters, Gkelis *et al.* [44] found compounds with the UV spectra typical of anabaenopeptins and anabaenopeptilides. In our environmental studies, the presence of anabaenopeptins in blue mussels was documented for the first time. The peptides were detected using MRM experiments and their structures were confirmed on the basis of mass fragmentation spectra of pseudomolecular ions. As *N. spumigena* does not produce anabaenopeptins with arginine in the exocyclic positions [45], the two peptides with *m/z* 851 (AP E) and 837 (AP B) must derive from other cyanobacteria—probably *Dolichospermum* spp., which was also present in the collected phytoplankton sample.

In the mussels, no spumigins or aeruginosins were detected, though these peptides were present in a cyanobacterial sample collected one month earlier from the same area in the Gulf of Gdańsk. This fact can be explained by lower amounts of the compounds in cyanobacteria bloom samples. We can also hypothesize that the process of depuration of these linear and more polar peptides through excretion and/or biotransformation is faster, and therefore, they have a lower tendency to accumulate in the exposed organisms. Assuming that the peak area in the MRM chromatogram roughly corresponded to the concentration of the compound, it can be observed that smaller mussels contained higher amounts of anabaenopeptins. A similar observation was made by Mazur-Marzec *et al.* [8] with respect to concentrations of NOD in two size classes of blue mussels from the Gulf of Gdańsk. These differences in the level of the peptides in mussels might be attributed to less effective filtration caused by undeveloped gill structure in the juvenile forms of the organisms [46].

The accumulation of NODs and APs was also unequivocally proven in the crustaceans exposed under laboratory conditions to the extract from a cyanobacteria bloom sample. In the organisms, SPU611 and AER587 were additionally detected. In the cyanobacterial extract, these two compounds belonged to the most abundant representatives of the linear peptides. In laboratory experiments, at constant exposure to the cyanobacterial extract, the crustaceans could apparently accumulate detectable amounts of SPU and AER. Differences in the profiles of peptides detected in crustaceans and in mussels can also result from various efficiencies of the metabolic processes in the exposed organisms. Bioaccumulation of compounds is always determined by the balance between the rate of their assimilation and losses due to the processes of biotransformation and excretion.

### 3.2. The Effect of Cyanobacteria Metabolites on Crustaceans

At high concentration of NOD, reaching 5.6 mg∙L^−1^ in an undiluted sample, over 90% of crustaceans used in our experiments died (Figure 1). This result corresponds to the data published by DeMott *et al.* [47]. For *Daphnia* and *Diaptomus birgei* treated with pure toxin, these authors determined LD_50_ values of NOD as 3.9–14.1 mg∙L^−1^ and 0.52–1.25 mg∙L^−1^, respectively. Such high concentrations of NOD have been occasionally measured in coastal waters of the Gulf of Gdańsk during the peak of cyanobacteria bloom (up to 42.3 mg∙L^−1^) [8]. However, even under the extreme cyanotoxin concentration, acute effects on zooplankton have not been observed in the sea. Probably, the patchy distribution of surface cyanobacteria accumulations, their quick dilution within water masses, and the vertical migration of zooplankton [48] reduce the risk of harmful effects. Moreover, laboratory experiments and analysis of long term data showed a positive effect of the Baltic cyanobacteria on the reproduction and development of copepods [11]. The small crustaceans used in our bioassays probably did not experience previous contact with *N. spumigena* and its metabolites. This could make the organisms more susceptible to harmful effects than the Baltic invertebrates, which are regularly exposed to toxic blooms of *N. spumigena*. It was proven that pre-exposure of zooplankton to toxic cyanobacteria enhanced their resistance to harmful bloom events [49]. In mussels and crustaceans, contact with cyanobacteria resulted in the induction of detoxication processes, including activation of glutathione S-transferase GST and glutathione peroxidase GPx [24,50,51,52].

The response of invertebrates to toxic cyanobacteria is also species-dependent. Marsálek and Blaha [5], using standard biotests, found *T. platyurus* to be more sensitive to crude extracts and fractions from *Microcystis* than *A. salina*. In our work, the sensitivity of the two species of crustaceans, *T. platyurus* and *A. franciscana,* towards *N. spumigena* was tested (Figure 2 and Figure 3)*.* In the eight-times diluted cell extract and in the spent medium, the percentage of dead *A. franciscana* organisms was higher than *T. platyurus*. The differences observed in our tests can be attributed to some extent to the fact that under the experimental conditions, *A. franciscana* accumulated higher amounts of *N. spumigena* metabolites than *T. platyurus.*

It has been often reported that toxic cyanobacteria or their crude cell extracts have a greater impact on tested organisms than a pure toxin [5,15]. Adverse effects were also revealed in crustaceans exposed to non-toxic cyanobacteria or microcystin (MC)-free fractions from cyanobacterial cells [53,54]. In the tests performed in our work, high mortality of *A. franciscana* was observed not only in the NOD-containing fraction III but also in the NOD-free fraction II, characterized by the presence of the highest number of peptides classified to SPUs and AERs. These peptides are known inhibitors of trypsin-like serine proteases [30,55,56]. APs belong to inhibitors of protein phosphatase [57]. Some of their variants are also active towards proteolytic enzymes such as trypsin, chymotrypsin, elastase, and carboxypeptidase-A [31,58,59]. Serine proteases and protein phosphatases are involved in many important physiological processes, and their deregulation by cyanobacterial metabolites may have adverse effects on the exposed organisms. Inhibitors of proteases were shown to be frequently present in cyanobacterial bloom samples from different water bodies [60,61], including the Baltic Sea [35].

We think that the observed reactions of the crustaceans to *N. spumigena* extracts could be a result of combined effects of different enzyme inhibitors produced by the microorganism*.* Inhibition of protein phosphatases PP 1 and PP 2A in *Daphnia* by MC-LR was revealed by DeMott and Dhawale [62]. The inhibitory effect of cyanobacterial metabolites on the activity of trypsin and chymotrypsin in *Daphnia* was reported in several published works [20,61,63,64,65,66]. The presence and significance of these enzymes in crustaceans were documented by Agrawal *et al.* [63] and Von Elert *et al.* [67]. Lower activity of proteolytic enzymes can lead to reduced food digestion in cyanobacteria grazers. In *Daphnia*, inhibition of protease caused lethal molting failure [18]. Among the cyanopeptides with a confirmed negative effect on crustaceans, there are cyanopeptolins [20,63,66], oscillapeptins [68], and microviridin J [18]. In addition, the experiments on *Daphnia magna* showed that microcin SF608, an aeruginosin variant, lowered the cellular concentration of microsomal and soluble glutathione-*S*-transferase—the enzyme involved in detoxication processes in plants and animals [69].

The negative consequences of exposure to cyanobacteria might be weaker in invertebrates from the Baltic Sea, which regularly experience cyanobacterial blooms. Von Elert *et al.* [66] revealed that the presence of protease inhibitors in the diet of *Daphnia* increased their tolerance to cyanobacteria.

## 4. Experimental Section

### 4.1. Sample Collection and Growth of Cyanobacteria

In the laboratory experiment, a monospecies, not an axenic culture, of the Baltic *N. spumigena* (strain CCNP1401) was used. The cyanobacterium was grown in a Z8 medium (without nitrogen), prepared using MilliQ water (PF system, Millipore, Molsheim, France) with NaCl added to produce a final salinity of 7 psu. *N. spumigena* culture was incubated for 21 days in growth chambers at 22 °C and irradiance of 20 μmol photons m^−2^∙s^−1^ with a 16:8 light:dark cycle. Then, it was filtered (100 mL) through Whatmann GF/C filters. In the bioassay, the crude extract from cells collected on day 21 of cyanobacteria culture and the spent media collected on progressive days (day 4, 8, 11, 15, 18 and 21) during the culture were used.

In addition, on 27 July, 2012, during a bloom composed of *N. spumigena* (55% biomass), *A. flos-aquae* (35%), and *Dolichospermum* spp. (10%), cyanobacterial biomass was collected with a plankton net (50 μm) from a station located in the Gulf of Gdańsk (54°28.8´ N; 18°39.0´ E; 20 m depth). Due to technical reasons, blue mussels (*Mytilus trossulus*) could be not collected from the same station earlier than 20 August 2012. The organisms were divided into two classes depending on their size: shell length (L) below 2 cm (L < 2 cm) and over 3 cm (L > 3 cm). The soft tissue was removed and lyophilized.

The schematic diagram presenting the setup of experiments conducted in this work is shown in Figure S6 (A,B).

### 4.2. Extraction and Fractionation of Cyanobacteria Metabolites

The extract from *N. spumigena* CCNP1401 cells collected on day 21 of the culture (1 g wet weight, w.w.) and the extract from the cyanobacterial bloom sample (482 mg dry weight, d.w.) were prepared in 90% methanol by grinding followed by sonication with a Bandelin Sonoplus HD 2200 (Bandelin, Berlin, Germany) equipped with MS72 (1 minute). After centrifugation (10,000 *g* for 15 min), the solvent was removed from the supernatant by rotary evaporation at 35 °C.

The extract from *N. spumigena* CCNP1401 cells was divided into two equal portions. One portion was tested in the crustaceans bioassay. The second part of the extract was dissolved in 1 mL of 30% methanol and fractionated by repeated injections (75 µL) into an analytical HPLC system (Agilent 1200, Agilent Technologies, Waldboronn, Germany) equipped with a fraction collector. The separation was performed on a Luna RP-18 column (3.0 mm × 150 mm; 3 μm). Gradient elution with the mobile phase A (5% acetonitrile in MilliQ water with 0.05% trifluoroacetic acid, TFA) and B (100% acetonitrile with 0.05% TFA) was used. The mobile phase was delivered at a flow rate of 0.5 mL∙min^−1^. Phase B was linearly increased from 30% to 40% in 20 min and then to 100% in 5 min. The column was washed with 100% phase B for 10 min, then the mobile phase composition was brought back to the initial conditions (70% B) in 3 min. The absorbance at 215, 238, 256, and 280 nm was monitored. Four fractions containing different classes of peptides were collected: I (0–3 min), II (3.1–7.7 min), III (7.8–10.0 min) and IV (10.1–15.0 min), and then evaporated to a dry residue.

### 4.3. Biological Tests

Two anostracan crustaceans were used to test the activity of metabolites produced by *N. spumigena* CCNP1401: *Thamnocephalus platyurus* (Thamnotoxkit F™) and *Artemia franciscana* (Artoxkit M™) (MicroBioTests INC, Gent, Belgium)*.* For this purpose, the crude cyanobacterial cell extract, fractions, and the spent media collected on different days of culture were analyzed. The bioassays were conducted according to the standard operating procedures supplied by the manufacturer. The cell extract and evaporated fractions were dissolved in 100 mL of crustacean incubation medium from the test kits. The mortality percentage was calculated by counting the number of dead organisms after 24-hour incubation at 25 °C.

In the crustaceans, the accumulation of cyanobacterial metabolites was also examined. In this experiment, the extract from the cyanobacterial bloom sample was dissolved in the incubation medium so that the initial concentration of nodularin was 1 mg∙L^−1^. The test organisms were exposed to the sample for 24 h in the dark, at 25 °C. They were then washed six times in a medium (without extract) and centrifuged (2500 *g* for 15 min).

### 4.4. Extraction of Cyanobacterial Peptides from Crustaceans and Mussels

*T. platyurus* and *A. franciscana* (50 mg d.w.) and blue mussels (500 mg d.w.) were homogenized and extracted with 5% acetic acid (3 mL per 100 mg) by 5-min probe sonication followed by 15-min bath sonication. After centrifugation, the supernatants were collected and washed with hexane (1 mL per 3-mL extract). The obtained samples were purified by solid phase extraction on 0.5 g (crustaceans) or 1 g (mussels) Sep-Pak Vac C18 cartridges (Waters, MA, USA). The fractions eluted with 90% methanol were centrifuged and analyzed by LC-MS/MS.

### 4.5. Chemical Analysis of Cyanobacterial Peptides

The content of cyanopeptides in the cyanobacterial bloom sample, in the fractions obtained from the *N. spumigena* CCNP1401 cell extract, and in the extracts from crustaceans and blue mussels was analyzed with LC-MS/MS. The system consisted of Agilent 1200 HPLC (Agilent Technologies, Waldboronn, Germany) and a hybrid triple quadrupole/linear ion trap mass spectrometer (QTRAP5500, Applied Biosystems, Sciex; Concorde, ON, Canada). Separation of cyanopeptides was performed on a Zorbax Eclipse XDB-C18 column (4.6 × 150 mm; 5 µm) (Agilent Technologies, Santa Clara, California, USA) using a mixture of 5% acetonitrile in water (phase A) and 100% acetonitrile (phase B), both containing 0.1% formic acid. The MS/MS system operated in a positive ion mode with TurboIonSpray (550 °C) voltage 5.5 kV. To detect cyanopeptides in the analyzed samples, multiple reaction monitoring experiments (MRM) at a collision energy of 50 eV and dwell time of 100 ms were performed. For nodularin, whose standard was available, analysis was conducted using the following transitions: 825 > 135 (quantifier), 825 > 227, and 825 > 163. The relative quantity of other cyanopeptides was assessed based on their peak areas in the MRM chromatograms. For a given peptide, the peaks corresponding to the transitions with the highest intensity were compared. In the analyses, the following transitions were used for spumigins (613 > 449, 255, 144, 84; 611 > 342, 165, 107, 84; 597 > 439, 342, 107), aeruginosin (587 > 266, 221, 136), anabaenopeptins (918 > 689, 339, 107; 916 > 856, 691, 107; 902 > 711, 495, 107; 900 > 488, 164, 107; 884 > 689, 511, 339, 164, 107; 868 > 711, 495, 84; 851 > 651, 201, 175; 837 > 637, 201, 175; 828 > 405, 120, 84; 824 > 667, 451, 164, 107; 808 > 651, 435, 148, 84) and for [Asp^3^] NOD (811 **>** 691, 389, 135). To confirm the structure of the peptides detected in the invertebrates, their enhanced ion product (EIP) spectra were acquired at a collision energy (CE) of 50 V. EIP mode was also used in the analyses of *N. spumigena* peptides in cell extracts, fractions, and the culture medium. Data acquisition and processing were accomplished using Analyst QS^®^ 1.5.1 software.

### 4.6. Statistical Analysis

Statistical analysis was performed using Microsoft Excel 2013 (v15.0). The results of assays performed with Thamnotoxkit F™ and Artoxkit M™ were considered to be significant when the crustaceans’ mortality did not exceed 10% in control variants, and was higher than 20% in the tested samples.

## 5. Conclusions

Our studies proved the accumulation of different classes of cyanobacterial peptides in two representatives of aquatic invertebrates: the crustaceans and blue mussels. We also showed that apart from nodularin, other *N. spumigena* metabolites might be active against *A. franciscana.* These results indicate that cyanobacterial toxicity to aquatic organisms is a complex phenomenon and the induced effects can be attributed to the activity of diverse metabolites, not only to the known toxins. As there are significant inter- and intraspecies differences in the metabolite profile and in the reaction to their exposure, the type and strength of the interactions between organisms are also expected to be strain-specific, with respect to both the cyanobacteria and the grazers.

## Supplementary Files

Supplementary File 1
