# Supplementary material for: Nodularia spumigena Peptides—Accumulation and Effect on Aquatic Invertebrates
Source: Toxins (Basel). 2015 Oct 30;7(11):4404–20. doi: 10.3390/toxins7114404 (PMC4663510; doi:10.3390/toxins7114404)
Supplement: Supplementary File 1 [file toxins-07-04404-s001.pdf]

Supplementary Information

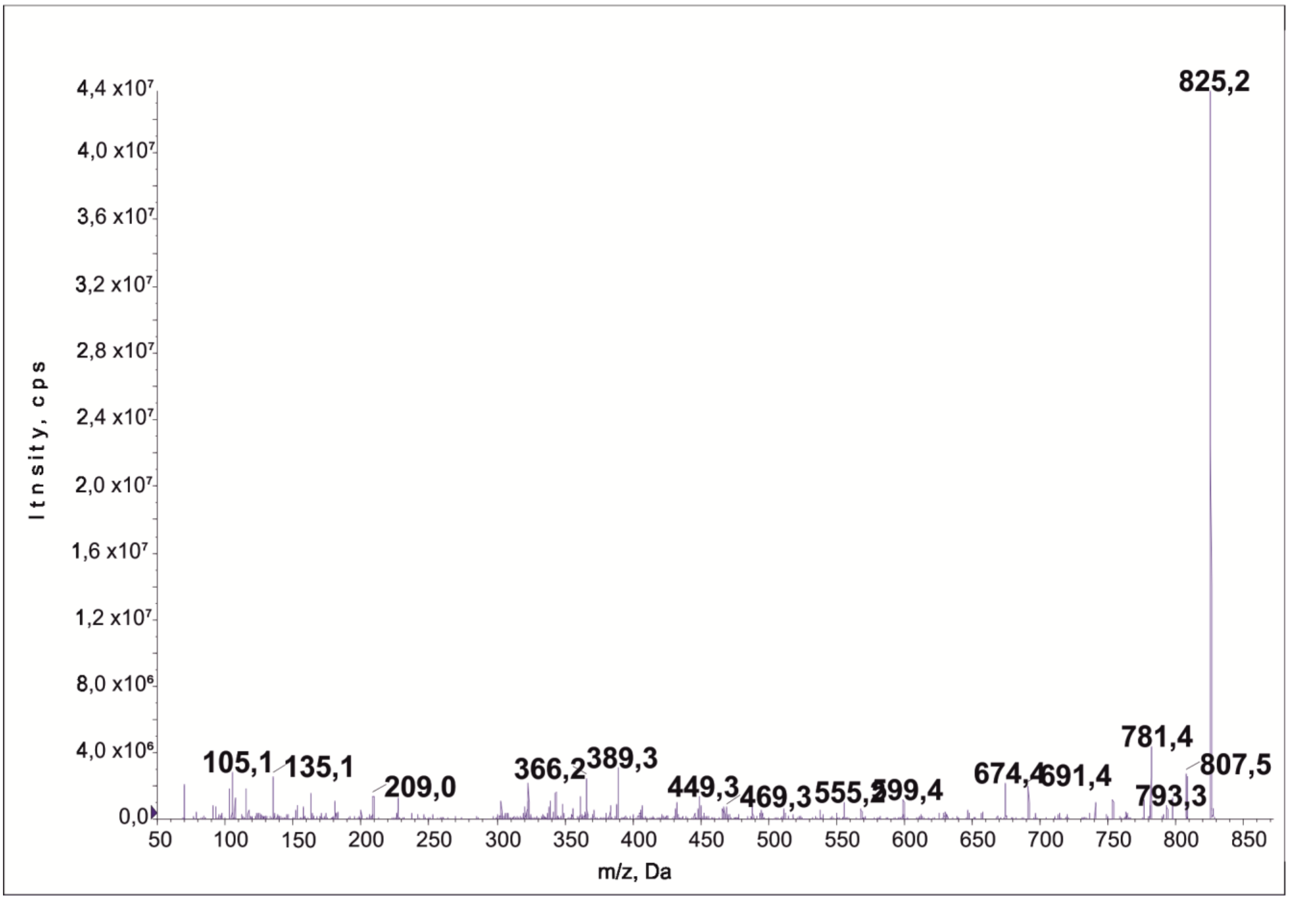

Figure S1. Fragmentation spectrum of nodularin detected in *Artemia franciscana* exposed to *Nodularia spumigena* extract. The peptide is characterized by pseudomolecular ion $[M+H]^+$ at *m/z* 825 and structure Cyclo[MeAsp-Arg-Adda-Glu-Mdhb].

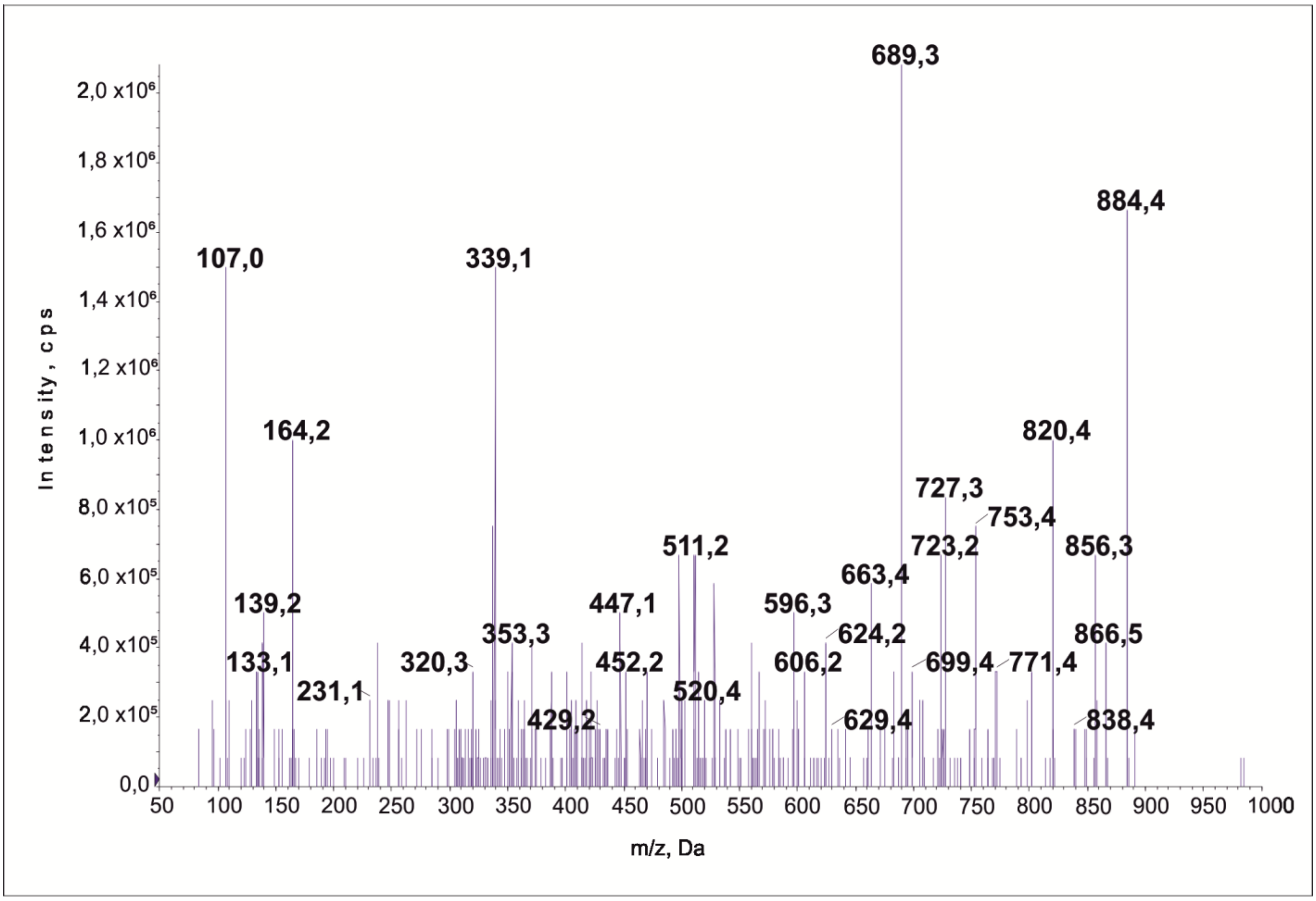

Figure S2. Fragmentation spectrum of anabaenopeptin AP884 detected in *Artemia franciscana* exposed to *Nodularia spumigena* extract. The peptide is characterized by pseudomolecular ion $[M+H]^+$ at *m/z* 884 and structure Ile-CO-[Lys-Val-Hph-MeHty-MetO].

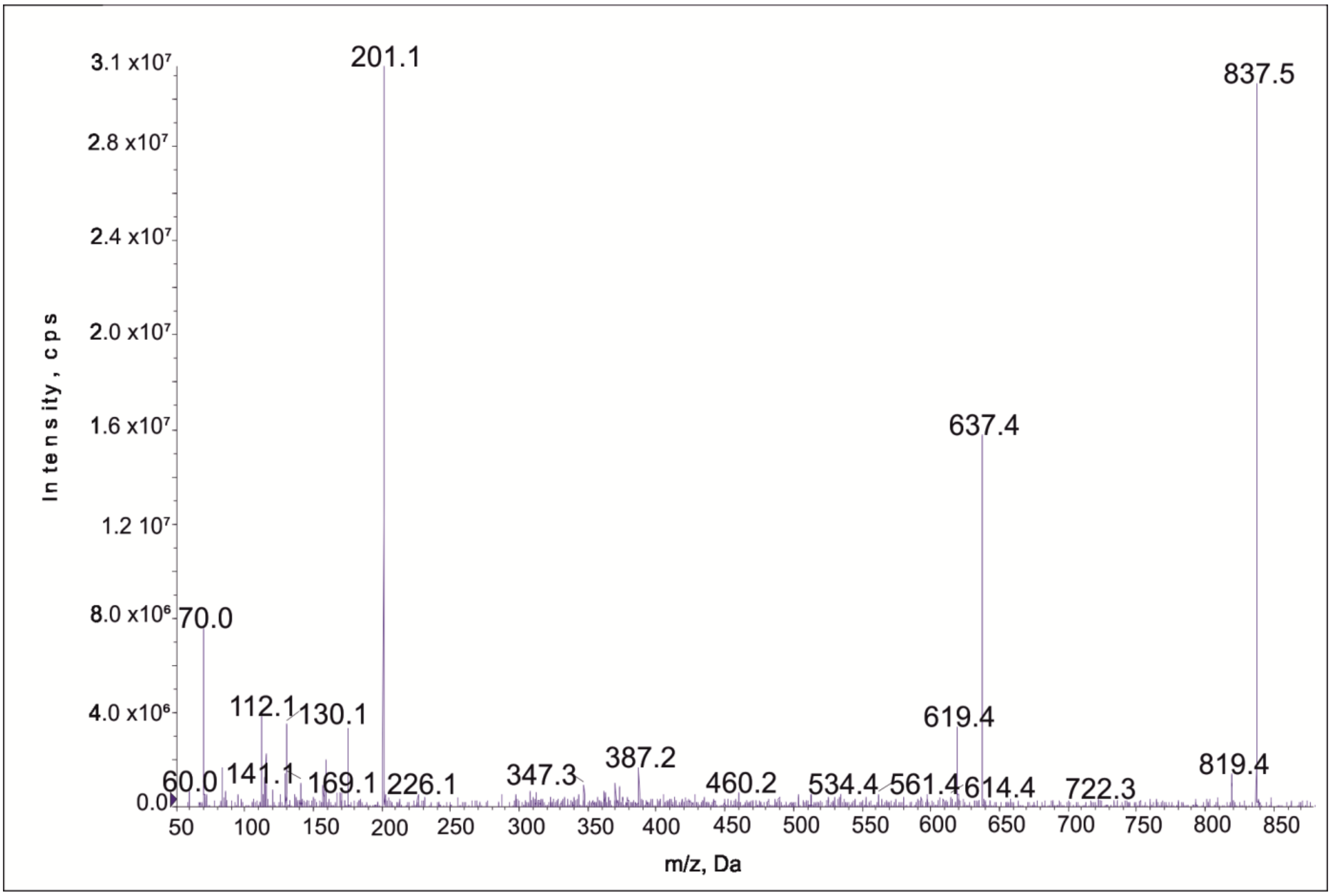

Figure S3. Fragmentation spectrum of anabaenopeptin B (AP837) detected in smaller mussels (<2 cm). The peptide is characterized by pseudomolecular ion $[M+H]^+$ at *m/z* 837 and structure Arg-CO-[Lys-Val-Hty-MeAla-Phe].

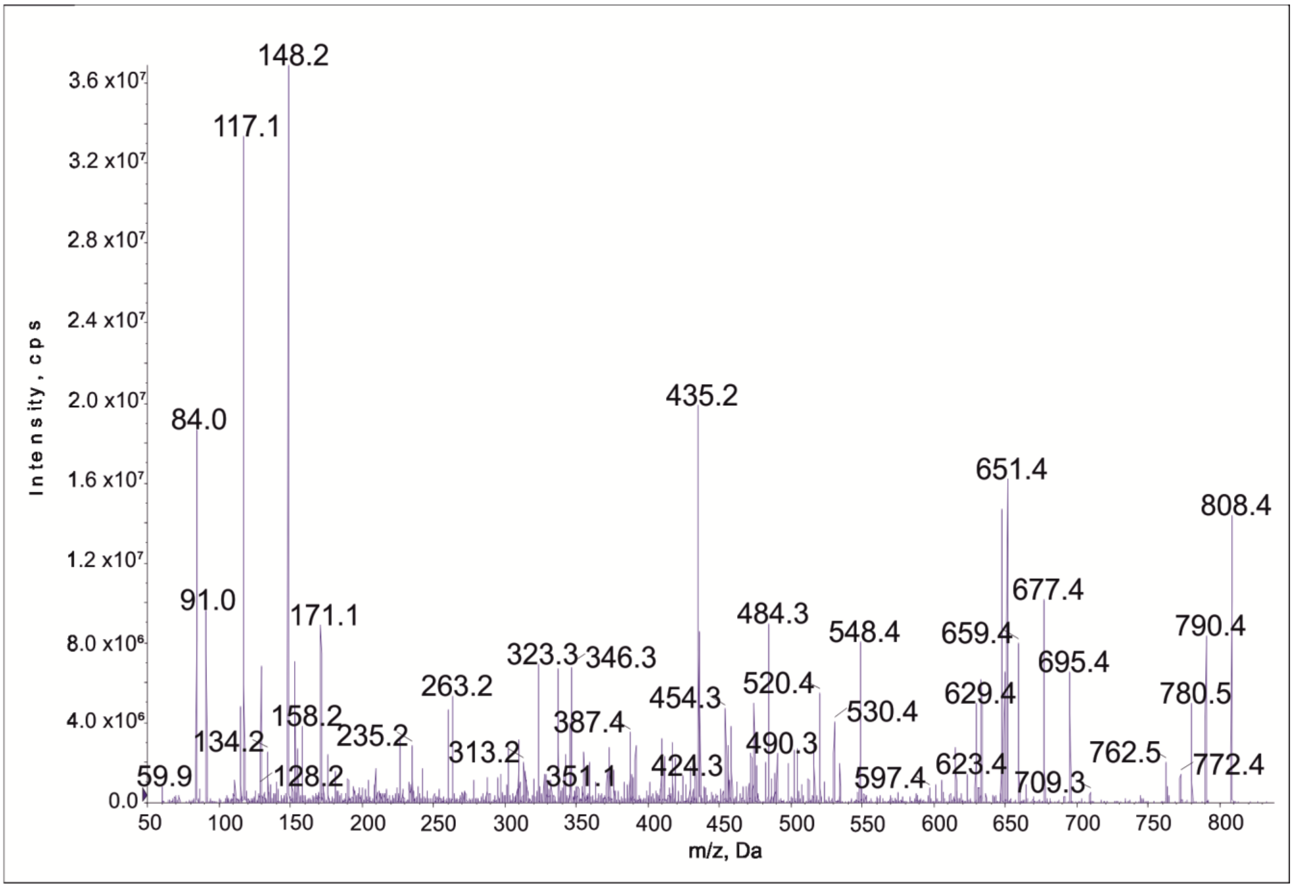

Figure S4. Fragmentation spectrum of anabaenopeptin AP808 detected in smaller mussels (<2 cm). The peptide is characterized by pseudomolecular ion [M+H]+ at m/z 808 and structure Ile-CO-[Lys-Val-Hty-MeAla-Hph].

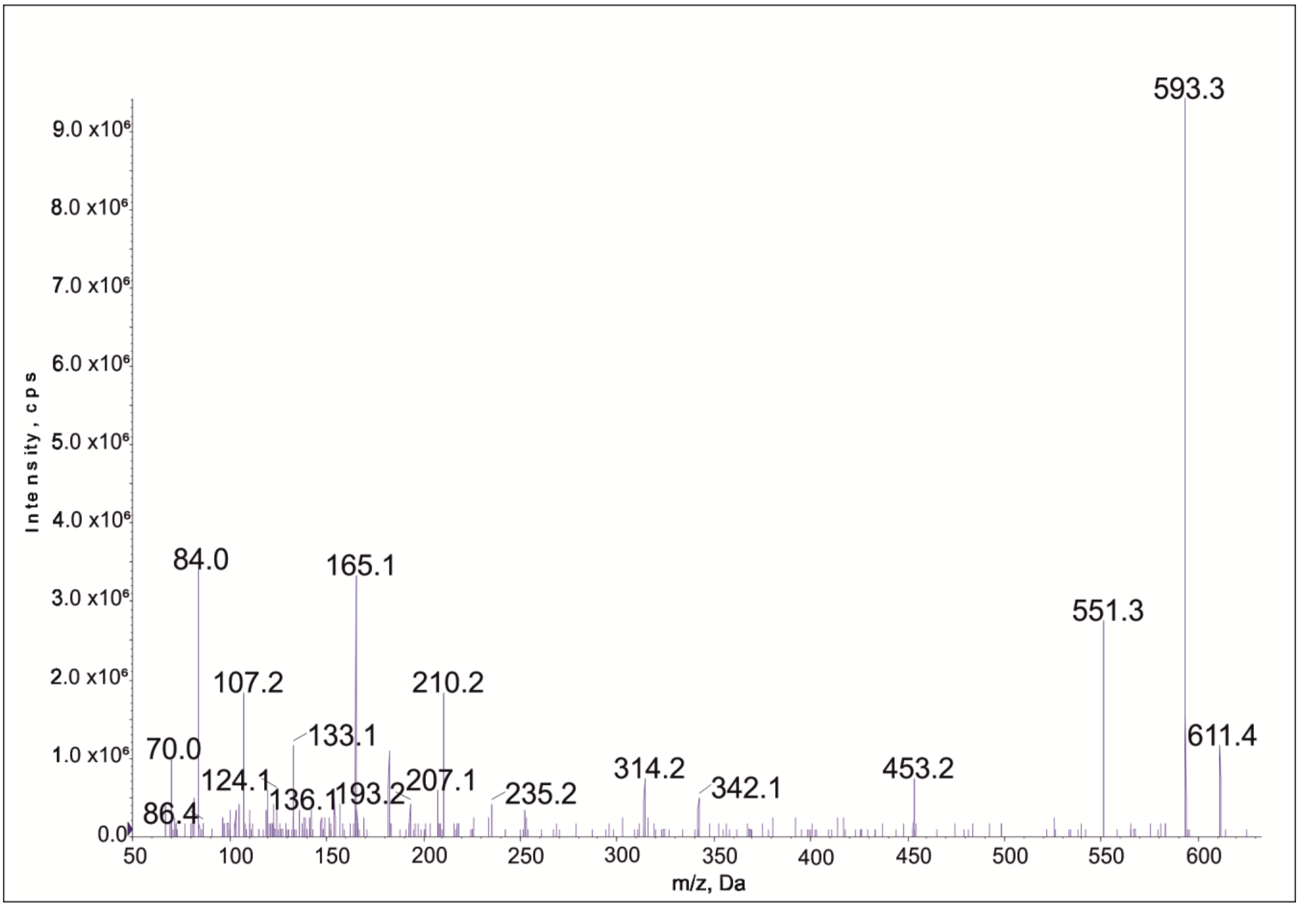

Figure S5. Fragmentation spectrum of spumigin SPU611 detected in *Artemia franciscana* exposed to *Nodularia spumigena* extract. The peptide is characterized by pseudomolecular ion $[M+H]^+$ at *m/z* 611 and structure Hpla-Hty-MePro-Argal.

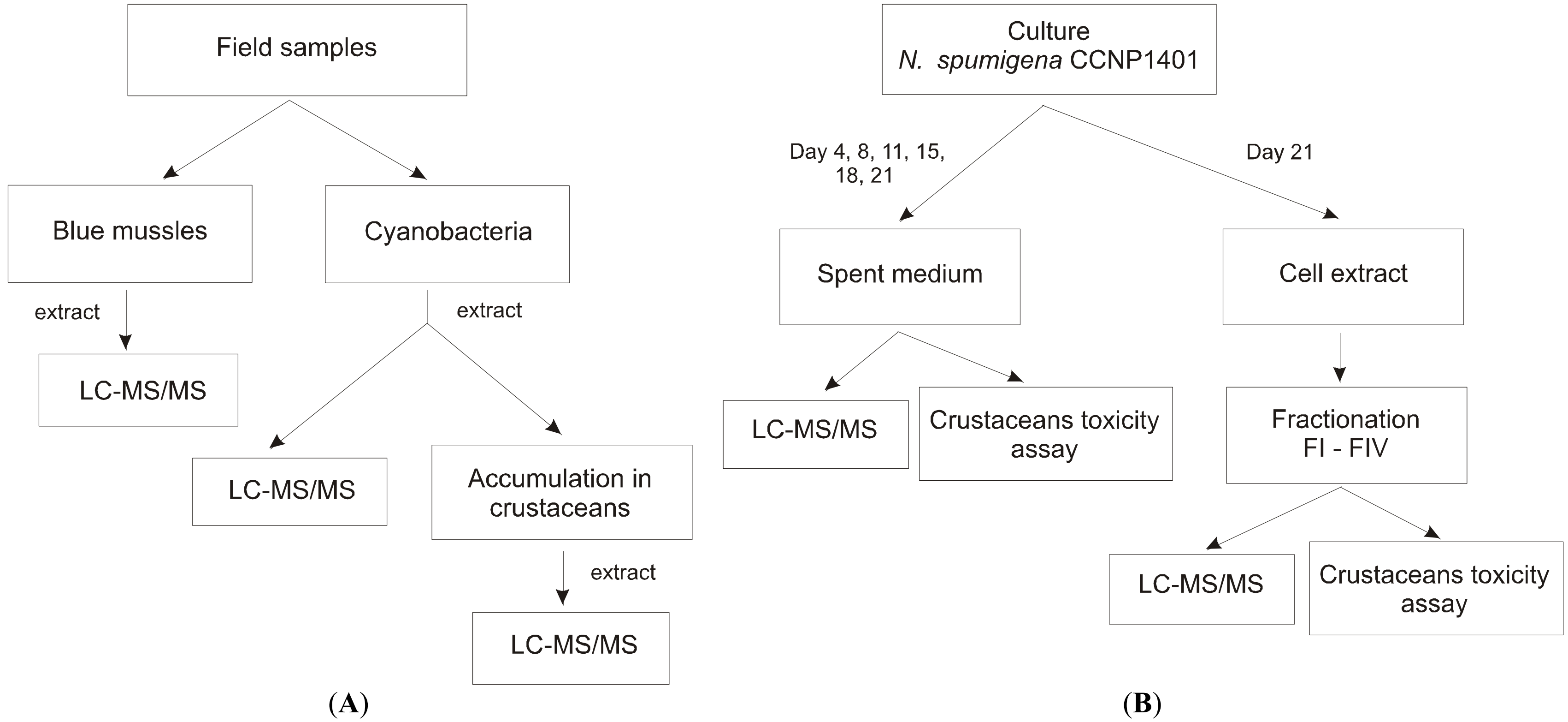

Figure S6. Schematic diagram of experimental setup: Accumulation of cyanobacterial peptides in blue mussels and crustaceans *Thamnocephalus platyurus* and *Artemia franciscana* (**A**); Effect of *Nodularia spumigena* cell extract and spent medium on the crustaceans (**B**).
